# Maternal depressive symptoms and children’s cognitive development: Does early childcare and child’s sex matter?

**DOI:** 10.1371/journal.pone.0227179

**Published:** 2020-01-10

**Authors:** Chantal Paquin, Sylvana M. Côté, Richard E. Tremblay, Jean R. Séguin, Michel Boivin, Catherine M. Herba

**Affiliations:** 1 Department of Psychology, Université du Québec à Montréal, Montréal, Québec, Canada; 2 Centre de Recherche du Centre Hospitalier Universitaire Sainte-Justine, Montréal, Québec, Canada; 3 Department of Social and Preventive Medicine, University of Montréal, Montréal, Québec, Canada; 4 INSERM U1219 Bordeaux Population Health Unit (BPH), University of Bordeaux, Bordeaux, France; 5 Departments of Psychology and Pediatrics, University of Montréal, Montréal, Québec, Canada; 6 School of Public Health, Physiotherapy and Population Science, University College Dublin, Dublin, Ireland; 7 Department of Psychiatry and Addictology, University of Montréal, Montréal, Québec, Canada; 8 School of Psychology, Laval University, Québec, Québec, Canada; Technion Israel Institute of Technology, ISRAEL

## Abstract

**Background:**

Maternal depressive symptoms (MDS) have been associated with poorer child cognitive development. Some studies have shown that childcare attendance moderates associations between MDS and child behavior problems, but we do not know if this is the case for children’s cognitive development. Furthermore, few studies have evaluated whether associations between MDS and child cognitive development differ for boys and girls at school entry.

**Methods:**

This study used data from a population-based cohort study (n = 1364) comprising well-validated measures of children’s cognitive development including academic readiness and language development in kindergarten and reading and mathematics achievement in first grade. Information on MDS was collected repeatedly from the child's age of 5 months to 5 years and on childcare from 5 months to 4.5 years. Moderation analyses were conducted to evaluate the differential associations of MDS with children’s outcomes depending on the type of childcare attended and the child’s sex.

**Results:**

Childcare type or child’s sex did not moderate associations between MDS and children’s cognitive outcomes except for MDS being associated with lower scores on reading achievement in first grade for girls with a very small effect size (sr^2^ = .003). Childcare attendance was associated with higher scores for children’s cognitive development, however these associations disappeared after adjusting for covariates including child, mother and family characteristics. Regardless of MDS and childcare type, boys had, even after adjusting for covariates, lower scores on academic readiness (sr^2^ = .029) and higher scores on mathematics achievement (sr^2^ = .004).

**Conclusions:**

Children’s cognitive development at school entry was more strongly associated with maternal education, children’s age in kindergarten and number of months of schooling in first grade than MDS. Contrary to associations between MDS and child behavior problems, childcare attendance did not moderate associations between MDS and children’s cognitive development at school entry.

## Introduction

Children’s cognitive development is a broad concept that includes academic readiness, language development, reading and mathematics achievement. Parental engagement such as taking the time to read, play, and discuss with one's child accounts for a large part of children's cognitive development [1–4]. Parenting practices such as parental sensitivity, warmth and responsiveness are also linked to cognitive development [4–7]. However, mothers with elevated depressive symptoms tend to be less inclined to read, play, or sing with their child [8]. For instance, studies have reported that depressed mothers ask fewer questions and provide fewer explanations to their child [9]. Even mothers presenting low levels of depressive symptoms tended to play less often with their child and provide less academic stimulation to their preschool child, such as working with numbers and colors, compared to mothers with no depressive symptoms [10]. They also tend to be less sensitive, to use more harsh parenting, to be more hostile and to express more anger towards their child than mothers with no depressive symptoms [5, 7, 11–13].

During the early childhood period (from birth to age 5 years), postnatal maternal depression had been negatively associated with children’s cognitive outcomes such as language and general cognitive development [14, 15]. These associations may be particularly salient in the early childhood period compared to middle childhood or adolescence given the increased dependence of the child on the mother. However, evidence for the association between maternal depressive symptoms (MDS) and children’s cognitive development at school entry is inconsistent, as described below. This is likely due to differences in the severity of maternal depression or MDS across the different studies, as well as the influence of other relevant variables that contribute to children’s cognitive development such as the amount of schooling the child has completed or their experience of childcare over the early childhood period as well as the contribution of maternal education.

This study aims to extend the knowledge on the associations between MDS and several measures of children’s cognitive development including academic readiness and language development in kindergarten and reading and mathematics achievement in first grade. We assess such associations within the context of a large-scale longitudinal study of children followed since infancy, while examining whether childcare during the early childhood period or child’s sex moderates these associations. We also examine whether MDS has a unique contribution to children’s cognitive outcomes over and above other contextual risk factors associated with MDS and children’s cognitive development, such as maternal education or socio-economic variables.

### Maternal depression and children’s cognitive development

Brennan and colleagues [16] reported a small negative association between MDS severity and chronicity, measured from pregnancy to 5 years of age, and children’s language development at age 5 years (N = 3,767). More recently, using data from the Quebec Longitudinal Study of Child Development (QLSCD), Ahun and colleagues [17] reported that chronic maternal depression over the child's age of 5 months to 5 years was associated with lower scores on language development (n = 1,073). Children's language development was assessed using the Peabody Picture Vocabulary Test-Revised (PPVT-R) and an average score was calculated based on assessments when the child was aged 5, 6 and 11 years old. Létourneau, Tramonte, and Willms [18] compared associations between different maternal depression symptom patterns during the early childhood period (never depressed, postpartum only, recurrently depressed, late onset) and language development for 10,033 children aged 4 and 5 years old. Recurrent and late onset depression increased children’s risk of poorer language development.

Few studies have more specifically examined associations between MDS and abilities associated with academic readiness and reading or mathematics achievement outcomes. Mensah and Kiernan [19] studied associations between MDS and teacher-assessed reading and mathematics performance in 5-year-old children. Elevated MDS were associated with poorer performance for children. The association was stronger for boys’ mathematics achievement and tended to be stronger for boys’ reading achievement compared to girls. However, MDS were not evaluated prior to age 5 years and thus were not taken into account in their analyses. Kersten-Alvarez and colleagues [20] compared school outcomes for five-year-old children whose mothers were diagnosed with a postpartum depression episode (n = 21) with children from a community sample (n = 113). They found that children of depressed mothers scored lower on abilities associated with peer social competence and school adjustment. They also reported sex-specific effects for poorer language development among girls (but not boys) of mothers who reported postpartum depression compared to a community sample. Comaskey and colleagues [21] examined associations between maternal depression and anxiety disorders and children’s academic readiness in kindergarten for 18,331 mother-child dyads. They found a very small association between recurrent maternal depression and anxiety disorders and children’s academic readiness that was largely mediated by family context (e.g., being a teen-mother, single parent, lower socio-economic status).

Furthermore, research investigating whether children’s sex moderates associations between MDS and children’s cognitive development has yielded mixed results with some research reporting that boys were more affected by MDS than girls [19, 22], while other studies have reported the opposite pattern of findings [20, 23].

Children exposed to recurrent and chronic symptoms of depression are considered to be at increased risk for poorer cognitive development [3, 24] and the studies outlined above support this finding. However, effect sizes for associations between MDS and children’s cognitive outcomes have generally tended to be small [16, 17], although they may vary depending on the severity and/or chronicity of MDS, the type of ability assessed in the child, the age at which it is assessed and the child’s sex [3, 14, 17–20, 22, 23, 25]. Furthermore, childcare attendance during the early childhood years could be another relevant factor contributing to children’s cognitive development.

### Childcare attendance and children’s cognitive development

Several childcare arrangements exist that take place within or outside the family home. They can be categorized under formal or regulated childcare (e.g., center-based, family-based childcare) and informal or unregulated childcare (e.g., care by family members other than a parent, such as grandparents, brother or sister, or a babysitter within the children’s home) childcare. Formal childcare is often reported to be of higher quality than informal childcare [26, 27].

Research indicates that for children from vulnerable backgrounds, such as those characterized by low income or low maternal education, childcare attendance during the early childhood years, particularly formal childcare attendance, is associated with better cognitive development [28–31]. Other studies have indicated that for children of mothers with MDS, attending formal childcare during the early childhood period has been associated with fewer emotional and behavioral difficulties compared to those children of mothers with MDS who do not attend childcare [32–34]. Yet, knowledge is lacking on whether childcare may also be linked to better cognitive development for those children of mothers with MDS. A child's experience of childcare attendance over the early childhood years may also in part explain the inconsistent associations reported to date between MDS and child outcomes at school entry.

Based on findings to date, we hypothesized that MDS would be negatively associated with children’s cognitive development, and that childcare attendance would modify these associations. More specifically, we expected that associations between MDS and children’s cognitive development outcomes would be less pronounced for children who attended formal childcare during the early childhood years compared to those remaining at home during this period. In addition, we aimed to test whether associations between MDS and children’s cognitive development differ between boys and girls.

## Methods

### Participants

This study was conducted within the context of the Quebec Longitudinal Study of Child Development (QLSCD) led by the Institut de la statistique du Québec. The participants were randomly selected from the Québec registry of births for children born between October 1997 and July 1998 to ensure that all children in the cohort would enter school in the same year (singleton births, N = 2120). Information regarding the child was collected over the early childhood period at the ages of 5 months, 1.5, 2.5, 3.5, and 4.5 years old and at the beginning of formal school such as kindergarten (i.e., age 6 years) and grade 1 (i.e., age 7 years). The person identified as being the most knowledgeable (PMK) about the child was asked to provide information regarding the child and their use of childcare during the early childhood period. We studied normally developing children living with their biological mother, and thus selected participants for whom a) the PMK was the biological mother at each timepoint, and b) the PMK did not report important child limitations (e.g. autism, mental handicap). Furthermore, children’s cognitive development may be higher than other children of the same age if they skipped a school year or it may be lower if they repeated a school year and thus did not learn new material. We therefore focused on children who entered the school system at the statutory school starting age (i.e., did not skip or repeat a school year) leaving a total of 2026 mother-child dyads from the initial QLSCD sample. From these children, data were available for MDS and childcare at three or more time-points for 1832 mother-child dyads. The analyses included between 1109 and 1364 mother-child dyads depending on the child outcome assessed. Informed written parental consent was obtained at each assessment. Ethics approval was obtained from the Institut de la statistique du Québec’s ethics committee.

### Maternal depressive symptoms (MDS) measure

MDS were assessed at 5 months, 1.5, 3.5 and 5 years using a shortened version of the Center for Epidemiologic Studies Depression Scale [35], which is a widely-used self-report measure to assess symptoms of depression in the general population [36]. Twelve of the 20 items were used at 5 months and 1.5 years whereas 6 items were included at 3.5 and 5 years. Each item assesses the frequency of a depressive symptom during the past week on a 4-point scale ranging from 0 (none) to 3 (all the time). The shortened version demonstrates good psychometric properties [37]. The scores were rescaled to range from 0 to 10 to ensure that all timepoints had the same total score even if the number of items differed between timepoints. A minimum of three assessment points was used to calculate a MDS average score from 5 months to 5 years to ensure that we were capturing more than postnatal depressive symptoms. Mothers with missing information on two or more time points were excluded from the study (as indicated in the description of study participants).

### Childcare measures

When children were aged 5 months, 1.5, 2.5, 3.5 and 4.5 years, mothers reported on whether their child attended childcare (i.e., none [mother care], center-based childcare, cared for in someone else's home by a relative or a non-relative, cared for in own home by a relative or a non-relative) and the intensity of use (number of hours per week). Furthermore, when the child was cared for in someone else's home by a family-member or a non-family member, the mother indicated if the person providing the care was licensed by the government or approved by a recognized agency (i.e., if it was a regulated family-based childcare). The childcare arrangement was reclassified into three categories: 1) maternal care, 2) formal childcare that included center-based and regulated family-based childcare, and 3) informal childcare. Children with missing information on two or more time points were excluded from the study (as indicated in the description of study participants). If the child spent 9 hours or less per week in any childcare arrangement, they were included in the category of maternal care for that time point [29, 38, 39]. The total time spent in formal and informal childcare arrangements was calculated respectively considering all measurement points over the full early childhood period. Based on the total time spent in childcare, three groups were formed. If the child’s total time spent in formal and informal childcare was null, the child was included in the maternal care group (n = 232, 17.0%). Otherwise, if the total time spent in formal childcare was greater than the time spent in informal childcare, the child was included in the formal childcare group (n = 632, 46.3%). If not, the child was considered to be part of the informal childcare group (n = 500, 36.7%).

### Cognitive development measures

#### Kindergarten measures

Academic readiness was evaluated using the Lollipop Test [40]. It is composed of four subtests: 1) identification of colors and shapes and copying shapes; 2) picture description, position, and spatial recognition; 3) identification of numbers and counting; 4) identification of letters and writing [40]. A global score was calculated using the mean of the four subtests. This test has good convergent validity with other measures of academic readiness [41].

Language development was measured using the PPVT-R in either English [42] or French [43]. The child is presented with a group of four pictures at a time. The child must point to the picture corresponding to the word said by the interviewer. The test includes up to 170 words presented in increasing order of difficulty. The total score corresponds to the sum of correctly identified items. This test is a good predictor of subsequent academic achievement [44].

#### First-grade measures

Mathematics achievement was evaluated using an adapted version of Okamoto and Case Number Knowledge Test (NKT) [29, 45, 46]. This test assesses if the child can count concrete objects presented to him/her, as well as the child’s knowledge of number sequences, sums, and differences. The test ends when the child has committed three consecutive errors. The raw score was used indicating the number of correct answers provided. This test shows good predictive validity on subsequent mathematics abilities [44, 47].

Reading achievement was measured using the decoding and reading comprehension subtests of the Kaufman Assessment Battery for Children (K-ABC) Achievement Scale [48]. The decoding subtest requires the child to identify letters and to read and pronounce words while the reading comprehension subtests evaluates if the child understands simple sentences they have read, by requiring them to act out their meaning (ex: touch your nose). This test has good psychometric properties in English [48] and in French [49].

### Covariates

Factors associated with childcare attendance such as maternal education, insufficient income, marital status, and children’s temperament were identified as covariates to reduce the effect of social selection bias [50, 51]. Through a literature review, we also identified potential confounding variables contributing to our outcomes of interest. These variables can be categorized as child, mother and/or family characteristics. Covariates were assessed at the first data collection (i.e., 5 months of age).

Child characteristics included if the child was ever breastfed or not [52], low birth weight (<2.5 kg) [53, 54], the child’s temperament (difficult temperament subscale of the Infant Characteristics Questionnaire)[55, 56], and the child’s birth order (first-born child or not). The association between birth order and academic achievement is controversial (see [57]) but has been reported by some studies [58]. Child characteristics also included children’s age at the time of test administration (in years) and an indication of the time spent in school (in months). Children’s age had been positively associated with child cognitive development. Older children tend to perform better than younger ones in kindergarten and first grade [59, 60]. Furthermore, the number of months a child has spent in school has been positively associated with measures of academic achievement [60, 61]. This effect is particularly important at school entry [62]. Since all children were tested in the same calendar year but not the same month during kindergarten or in first grade, we calculated a variable indicating the number of months of schooling at the particular school level.

Maternal characteristics included the mother’s age at the child’s birth [63], maternal education [64], and parenting style [2]. Education was reported using a categorical indicator designating mothers without a high school degree, or those with either a high school, post high school or university degree. Mothers reported on their parenting style using the Parental Cognitions and Conduct Toward the Infant along 4 dimensions: perception of self-efficacy, perception of parental impact, coercive parenting, and overprotective parenting [65]. Perception of self-efficacy and perception of parental impact evaluate mothers’ beliefs about their role as a parent while coercive and overprotective parenting evaluate mothers’ behavioral tendencies. We evaluated these 4 dimensions separately.

Family characteristics included family structure (i.e., single-parent family or not) and insufficient income. Insufficient income (yes or no) is an index taking into account the size of the household and the region where it is located (e.g., urban versus rural, population density) based on guidelines set by Statistics Canada [66].

Only those variables that were significantly correlated with at least one of the academic outcomes were included in the final analyses. These variables included: mother’s age at childbirth, children’s age at test administration, number of months of schooling in kindergarten or grade 1 (depending on when the outcome was assessed), maternal education (three dummy variables created using no high school degree as a reference), parenting measures (subscale perceived impact and subscale overprotection), income status (insufficient or not), the children’s birth order (first-born child or not), birth weight (less than 2.5 kg or not), whether the child was breastfed or not and whether or not he/she lived in a single-parent family.

### Data preparation and statistical analysis plan

Data were analyzed using SPSS for windows (version 25.0; SPSS, Inc., Chicago, IL, USA). First, we computed descriptive statistics for the sample. The mean scores for children who took the PPVT-R test in French were significantly higher compared to those who completed the test in English. The PPVT-R score was thus transformed into Z scores separately for each version of the test (i.e., French and English) and then combined for the analyses. The same was done for the K-ABC scores (for the grade 1 assessment of reading ability). We also standardized the MDS score to facilitate the interpretation of results. Furthermore, we computed descriptive statistics and compared mother-child dyad characteristics in relation to their childcare attendance (i.e., maternal care, formal childcare, informal childcare).

Second, we evaluated the percentage of missing data on the identified covariates to be between 0 and 3.7%. According to Tabachnick and Fidell [67], in the case of large data sets with less than 5% of the participants having missing data, the different procedures for handling missing data provide very similar results. We used the SPSS multiple imputation procedure to create a single imputation data set using the full sample (N = 2120) and added auxiliary variables (family functioning and pregnancy length in weeks) correlating with our covariates. Family functioning was measured at 5 months using the McMaster Family Assessment Device [68]. The McMaster Family Assessment evaluates family functioning along different dimensions such as problem solving, communication, parenting roles, emotional receptivity and participation, and behavioral control [68]. A total score was calculated as described by Desrosiers, Boivin, and Des Grosseilliers [69]. A lower score indicates better family functioning.

Third, separate moderation analyses (two-way interactions) were conducted for each moderator and child outcome to evaluate the potential differential association between MDS and children’s outcomes depending on 1) type of childcare and 2) the child’s sex. We used a hierarchical regression approach as recommended by Hayes and Montoya [70] to test moderation with a multi-categorical moderator. We evaluated two different models: the first one included the main predictors (MDS, child’s sex, and childcare type) and children’s age at test administration as a covariate to focus on the central variables of interest and the second model including the predictors and all covariates (to better understand the contribution of MDS versus other contextual factors).

Finally, we used weights to ensure our study sample was comparable to the targeted population (n = 2026). Study participants (n = 1364) were compared along baseline characteristics with those who were not included in the analyses (n = 662) due to missing data on MDS, childcare type or child outcome. The characteristics that differed significantly between the two groups were then used to compute the probability for each mother-child dyad to be included in our analyses [71]. Each mother-child dyad was attributed a weight that was inversely proportional to the probability to be included in our analyses.

## Results

### Descriptive statistics

The mothers in this study were on average 28.93 years old at their children’s birth. Further data describing the sample are reported in Table 1. We also calculated the proportion of mothers with elevated MDS. To approximate the cut-off of 16 (out of 60) on the full Center for Epidemiologic Studies Depression (CES-D) Scale, we used a threshold of 2.67 out of 10 for our rescaled measures [17, 72]. At each time point between 14% and 18% of mothers reported elevated MDS which corresponds to prevalence rate of maternal depression reported in other studies [73, 74].

**Table 1:** Descriptive data.

Mother Characteristics	N	Mean (SD)	Minimum	Maximum
Average depressive symptoms (5 months to 5 years)	1364	1.37 (1.12)	0	7.37
Mother age at child birth (year)	1364	28.94 (5.17)	15.98	44.17
Parenting: overprotection	1338	5.30 (2.42)	0	10
Parenting: perceived impact	1337	8.47 (1.75)	0	10
Maternal education		%		
No high school	219	16.10%		
High school	338	24.80%		
Post high school	409	30.00%		
University	398	29.20%		
Child measures in kindergarten		Mean (SD)	Minimum	Maximum
Academic readiness (N = 1137)	1137	14.45 (1.74)	3	17.25
Age at test administration (year)	1137	6.23 (.25)	5.6	6.8
Number months in kindergarten	1137	5.12 (.88)	1	9
Language development (N = 1109)	1109	80.25 (16.80)	12	130
Age at test administration (year)	1109	6.23 (.25)	5.8	6.8
Number months in kindergarten	1109	5.12 (.90)	3	8
Child measures in first grade		Mean (SD)	Minimum	Maximum
Reading achievement (N = 1321)	1321	22.67 (10.50)	0	47
Age at test administration (year)	1321	7.14 (.26)	6.7	7.7
Number months in first grade	1321	3.94 (.78)	3	8
Mathematics achievement (N = 1364)	1364	19.67 (3.82)	6	27
Age at test administration (year)	1364	7.14 (.26)	6.7	7.7
Number months in first grade	1364	3.95 (.78)	3	6
Child Characteristics		%		
Sex (girls)	715	52.40%		
Birth weight < 2500 g	45	3.30%		
Breastfed (yes)	987	72.40%		
Birth order—first	597	43.80%		
**-** 2^nd^ or more	767	56.20%		
Family Characteristics		%		
Insufficient income	271	19.90%		
Single-parent family	88	6.50%		

Data courtesy of Institut de la statistique du Québec.

One-way analyses of variance (ANOVA) type II and chi-square analyses were performed to compare participants’ characteristics depending on their childcare attendance (i.e., maternal care, formal childcare, informal childcare). Significant statistical differences were found for the MDS average score during the early childhood period (*F*(2, 1361) = 8.62, *p* < .001), mothers' overprotective parenting (*F*(2, 1335) = 8.52, *p* < .001), maternal education (χ^2^(6, N = 1364) = 75.01, *p* < .001), single-parent family (χ^2^(2, N = 1362) = 7.37, *p* = .03), insufficient income (χ^2^(2, N = 1350) = 54.14, *p* < .001) and child birth order (χ^2^(1, N = 1364) = 26.91, *p* < .001). No differences were found for child’s sex, low birth weight, and breastfeeding (all *p*s > 0.05). As reported previously by Geoffroy et al. (2012) using this sample, Bonferroni post-hoc analyses indicated that children who did not attend childcare during the early childhood years were more likely to be the first-born child of the family, to have less-educated mothers, to have mothers scoring higher on overprotective parenting, and to come from families with insufficient income compared to children who attended formal or informal childcare in the early childhood period. They were also more likely to be from a single-parent family or to have a mother reporting higher MDS compared to children attending informal childcare. Mothers of children attending informal childcare reported significantly fewer MDS during the early childhood period than mothers of children not attending childcare or those attending formal childcare.

Mother-child dyads included in this study (n = 1364) were compared along baseline characteristics with those who were not included in the analyses (n = 662). Mothers of excluded dyads were more likely to be immigrants (14.7% vs. 4.9%; χ^2^(1, N = 2026) = 56.81, *p* < .001), to report higher MDS at 5 months (*F*(1, 2021) = 6.17, *p* = .01), higher scores on overprotective parenting (*F*(1, 1957) = 8.38, *p* < .01), and lower scores on perceived impact (*F*(1, 1955) = 11.95, *p* = .001). The excluded dyads were also more likely to report insufficient income (32.9% vs. 20.1%; χ^2^(1, N = 1992) = 38.80, *p* < .001), to be a single-parent family (11.1% vs. 6.5%; χ^2^(1, N = 2020) = 12.98, *p* < .001), and to have a boy (57.8% vs. 47.8%; χ^2^(1, N = 2026) = 19.94, *p* < .001), compared to dyads in the final sample. The two groups also differed on the highest maternal education level, with fewer mothers having a university degree (20.7% vs. 29.2%) and more mothers with no high school diploma (22.4% vs. 16.1%) among the excluded participants. The two groups were however similar on mothers’ age at childbirth, birth order and the proportion of children with low birth weight (all *p*s > 0.5).

Hierarchical linear regression analyses were conducted for each of the child outcomes, in kindergarten and first grade, to evaluate the potential differential associations between MDS and children’s outcomes depending on 1) childcare type and 2) the child’s sex separately. Two different models were analyzed for each moderator. Model 1 included MDS, child’s sex, and childcare type as predictors. Since we used raw scores for the children’s outcomes, we also included the child’s age at test administration as a covariate. Model 2 included MDS, child’s sex, childcare type as predictors and all covariates (children’s age at test administration, number of months of schooling in kindergarten or grade 1, maternal education, mothers’ perceived impact and overprotection, insufficient income, children’s birth order (first-born child or not), low birth weight (less than 2.5 kg or not), whether the child was breastfed or not, mother’s age at child’s birth, and whether the child lived in a single-parent family or not). All the regression analyses were conducted with and without weights and provided similar results, thus only the latter are reported here. Results for the main effects of variables (i.e., without interaction terms in the analyses) and the results of analyses testing moderation (i.e., interaction terms) are reported in Table 2 for kindergarten outcomes and Table 3 for first grade outcomes. Effect sizes for individual predictor variables were evaluated using the squared semi partial correlation (sr^2^) where 100*sr^2^ represents the percentage of variance explained by this predictor [75]. Semi partial correlation can be used to indicate small (.01), medium (.09) and large (.25) effect sizes [76].

**Table 2:** Moderation analyses of MDS x Childcare type and MDS x child’s sex for kindergarten outcomes.

	Kindergarten							
	Academic readiness (Lollipop)				Language development (PPVT-R)			
	*n* = 1137				*n* = 1109			
	Model 1		Model 2		Model 1		Model 2	
	β sr^2^		β sr^2^		β sr^2^		β sr^2^	
Covariates								
Child.s age	.12***	0.015	.16***	0.023	.12***	0.014	.16***	0.023
# months in grade			-.05^t^	0.002			-.05^t^	0.002
High school			.13**	0.008			.03	0.000
Post high school			.17***	0.012			.08	0.003
University			.23***	0.018			.19***	0.012
Perceived impact			.11***	0.011			.17***	0.026
Overprotection			.01	0.000			-.08*	0.005
Insufficient income			-.07*	0.004			-.12**	0.010
Single-parent family			-.02	0.000			-.00	0.000
Birth order (1st)			-.10**	0.008			-.12***	0.013
Low birth weight			-.08**	0.007			-.03	0.001
Mother's age			.04	0.001			-.01	0.000
Breastfed			.03	0.001			.00	0.000
Predictors								
MDS	-.04	0.002	.02	0.001	-.11***	0.012	-.02	0.000
Formal childcare	.12**	0.007	.03	0.000	.05	0.001	-.07^t^	0.002
Informal childcare	.15***	0.012	.07^t^	0.002	.10*	0.005	-.01	0.000
Child's sex (boy)	-.17***	0.028	-.17***	0.029	-.01	0.000	-.01	0.000
R^2^ change		.057		.142		.035		0.152
F	*F*(5,1131)	13.64***	*F*(17,1119)	10.86***	*F*(5,1103)	7.91***	*F*(17,1091)	11.48***
Moderator: childcare type								
MDS x formal care	.05	0.001	.04	0.000	.08	0.002	.01^t^	0.002
MDS x informal care	.00	0.000	.01	0.000	.01	0.000	.04	0.003
R^2^ change		.001		.000		.002		0.002
F	*F*(2,1129)	0.68	*F*(2,1117)	0.32	*F*(2,1101)	1.36	*F*(2,1089)	1.58
Moderator: child's sex								
MDS x child's sex	.06	31.000	.04	0.001	.06	0.001	.04	0.001
R^2^ change		.001		.001		.001		0.001
F	*F*(1,1130)	1.12	*F*(1,1118)	0.70	*F*(1,1102)	1.15	*F*(1,1090)	0.69

^t^p ≤ .10

*p ≤ .05

**p ≤ .01

***p ≤ .001.

Data courtesy of Institut de la statistique du Québec.

Note: standardized βs are reported.

**Table 3:** Moderation analyses of MDS x Childcare type and MDS x child’s sex for first grade outcomes.

	First grade							
	Reading Achievement (K-ABC)				Mathematics Achievement (NKT)			
	*n* = 1321				*n* = 1364			
	Model 1		Model 2		Model 1		Model 2	
	β sr^2^		β sr^2^		β sr^2^		β sr^2^	
Covariates								
Child's age	.08**	0.007	.08**	0.005	.08**	0.007	.07**	0.005
# Months in grade			.16***	0.023			.17***	0.027
High school			.21***	0.022			.10**	0.004
Post high school			.27***	0.030			.16***	0.011
University			.33***	0.038			.25***	0.022
Perceived impact			.09**	0.007			.05^t^	0.002
Overprotection			.00	0.000			-.03	0.001
Insufficient income			-.04	0.001			-.11***	0.009
Singl-parent family			-.03	0.001			.01	0.000
Birth order (1st)			-.06*	0.003			-.02	0.000
Low birth weight			.01	0.000			-.03	0.001
Mother's age			-.02	0.000			.04	0.001
Breastfed			.00	0.000			.01	0.000
Predictors								
MDS	-.08**	0.007	-.02	0.001	-.06*	0.004	.00	0.000
Formal childcare	.08^t^	0.003	-.03	0.000	.10**	0.005	.00	0.000
Informal chidlcare	.12**	0.007	.02	0.000	13***	0.008	.04	0.001
Child's sex (boy)	-.01	0.000	-.01	0.000	.06*	0.004	.06*	0.004
R^2^ change		.023		.127		.024		.129
F	*F*(5,1315)	6.13***	*F*(17,1303)	11.14***	*F*(5,1358)	6.66***	*F*(17,1346)	11.76***
Moderator: childcare type								
MDS x formal care	-.02	0.000	.01	0.000	.05	0.001	.07	0.001
MDS x informal care	-.03	0.000	.02	0.000	.02	0.000	.04	0.001
R^2^ change		.000		.000		.001		.001
F	*F*(2,1313)	0.19	*F*(2,1301)	0.02	*F*(2,1356)	0.45	*F*(2,1344)	1.02
Moderator: child's sex								
MDS x child's sex	.14**	0.006	.11*	0.003	.10*	0.003	.07	0.000
R^2^ change		.006		.003		.003		.002
F	*F*(1,1314)	7.65**	*F*(1,1302)	5.19*	*F*(1,1357)	4.29*	*F*(1,1345)	2.50

^t^p ≤ .10

*p ≤ .05

**p ≤ .01

***p ≤ .001.

Data courtesy of Institut de la statistique du Québec.

Note: standardized βs are reported.

#### Moderator: Childcare type

Analyses for model 1 demonstrate no moderation effect of childcare type for any of the child outcomes. We thus interpreted the main effect of childcare type. Attending formal childcare was associated with better child outcomes for academic readiness in kindergarten and mathematics achievement in first grade with small effect sizes varying from .005 to .007. Attending informal childcare was associated with better children’s outcomes for academic readiness and language development in kindergarten, as well as for reading and mathematics achievement in first grade with effect sizes varying from .005 to .012.

Analyses for model 2 (fully adjusted model) demonstrate no moderating effect of childcare type for any of the child outcomes. Furthermore, after adjusting for covariates, attending formal or informal childcare was no longer significantly associated with children’s outcomes.

#### Moderator: Child’s sex

Analyses for model 1 demonstrate that the child’s sex moderated the associations between MDS and reading achievement in first grade (*F*(1, 1314) = 7.65, *p* < .01) and mathematics achievement in first grade (*F*(1, 1357) = 4.29, *p* = .04). A simple slope analysis indicated that MDS was associated with reading achievement (β = -.15, *p* < .001) and mathematics achievement (β = -.45, *p* < .01) for girls but not for boys (β = .00, *p* = .93 and β = -.02, *p* = .91 respectively). Child’s sex did not moderate the associations between MDS and academic readiness and MDS and language development in kindergarten (all *p*s > 0.05). We thus interpreted the main effect of MDS and child’s sex. Elevated MDS were associated with lower scores on language development (β = -.11, *p* < .001) but were not associated with academic readiness in kindergarten, regardless of child’s sex. Child’s sex was associated with academic readiness indicating that boys tended to have lower scores than girls (β = -.17, *p* < .001) with an effect size of .029. Child’s sex was not associated with language development (β = -.01, *p* = .85).

With model 2 (fully adjusted), child’s sex continued to moderate the association between MDS and reading achievement in first grade (*F*(1, 1302) = 5.19, *p* = .02). A simple slope analysis confirmed that MDS were associated with reading achievement for girls (β = -.08, p = .03) but not for boys (β = .04, p = .31). For girls, there was a decrease of .08 standard deviation (SD) in reading achievement for each increase of 1 SD of MDS. Child’s sex no longer moderated the association between MDS and mathematics achievement in first grade (*F*(1, 1345) = 2.49, *p* = .11). Furthermore, MDS were not associated with mathematics achievement (β = -.00, *p* = .96), regardless of the child’s sex. However, boys had higher mathematics achievement scores (β = .06, *p* = .02), with an effect size of sr^2^ = 0.004. With regard to other child outcomes, MDS were no longer associated with language development (β = -.02, *p* = .42) and were not associated with academic readiness (β = .02, *p* = .40), irrespective of child’s sex. Furthermore, boys had lower scores on academic readiness (β = -.17, *p* < .001) with a small to medium effect size (sr^2^ = 0.029). No sex differences emerged for language development (β = -.01, *p* = .81).

#### Probing the contribution of covariates

The results of model 2 for the different outcomes indicated that children’s age at test administration was positively associated with all children’s outcomes with .005 < sr^2^ < .023. Contrary to our expectations, the number of months in kindergarten was not associated with academic readiness or language development. As expected, the number of months in first grade was positively associated with reading and mathematics achievement with an effect size of .023 and .027 respectively. Higher maternal education was associated with higher scores for all outcomes (.011 < sr^2^ < .037 for a university degree). Perceived maternal impact was associated with higher scores on academic readiness, language development and reading achievement (.007 < sr^2^ < .026). Being the first-born was also associated with a small decrease in scores for academic readiness, language development, and reading achievement (.003 < sr^2^ < .013). Insufficient income was associated with lower academic readiness, language development, and mathematics achievement in first grade (.008 < sr^2^ < .013).

#### Supplementary analyses

To ensure that our results were not due to the way of operationalizing the variable childcare type, we ran additional analyses. First, we slightly adapted the variable childcare type to also consider childcare arrangements of less than 10 hours per week. Thus, the total time spent in formal and informal childcare arrangements respectively was calculated. If the time spent in formal childcare was greater than the time spent in informal childcare, the child was included in the formal childcare group, otherwise the child was included in the informal group. If the child did not attend formal or informal childcare, he/she was included in the maternal care group. Using this modified childcare type variable, we did not find that childcare type moderated associations between MDS and children’s cognitive outcomes.

Second, to capture the intensity of childcare attendance (i.e. part-time versus full-time attendance), we created a new categorical variable to differentiate between children not attending childcare, children attending formal childcare less than 20 hours per week on average, children attending formal childcare more than 20 hours per week on average, children attending informal childcare less than 20 hours per week on average, children attending informal childcare more than 20 hours per week on average. Again, the same pattern of results was found; childcare did not moderate associations between MDS and children’s cognitive outcomes.

Third, we further probed the moderating role of childcare by testing whether childcare intensity of use (rather than the type per se) moderated associations between MDS and children’s cognitive outcomes. Previous studies have reported that childcare intensity of use attenuated associations between lower socioeconomic status and children’s cognitive outcomes [30] and between a more chaotic home environment and language development [77]. Based on the intensity of use reported at each time point (as described in the childcare measures section above where mothers reported on the number of hours per week in formal and informal childcare over the early childhood period), the total time spent in childcare was calculated for each child. The total time spent was averaged over the number of time points with childcare information (to make the information comparable if some time points were missing). The same models described above (e.g., model 1 with basic predictors and child age; and model 2 with all covariates) were evaluated using childcare intensity of use as a moderator (instead of childcare type).

Results for model 1 indicated that childcare intensity of use did not moderate associations between MDS and children’s cognitive outcomes. We thus interpreted the main effect of childcare intensity of use. Childcare intensity of use was associated with better academic readiness and language development in kindergarten, and better reading and mathematics achievement in first grade with effect sizes varying from .011 to .020. Results for model 2 (fully adjusted model) did not demonstrate a moderating effect of childcare intensity for any of the child outcomes. Furthermore, after adjusting for covariates, childcare intensity of use was no longer significantly associated with children’s cognitive outcomes.

## Discussion

We hypothesized that associations between MDS and children’s cognitive development at school entry would be less pronounced for children attending formal childcare compared to those who did not attend childcare during the early childhood years. Contrary to our expectations, we found that attending formal or informal childcare did not moderate associations between MDS and children’s cognitive outcomes at school entry. We also aimed to contribute to the discussion on the differential associations between MDS and children’s cognitive outcomes at school entry in relation to the child's sex. Higher MDS were associated with lower scores on reading achievement in first grade for girls, although the effect size was very small. Child’s sex did not moderate associations between MDS and any of the other children’s cognitive outcomes. Finally, we found that boys had poorer academic readiness in kindergarten and better mathematics achievement in first grade although effect sizes were quite small.

Childcare attendance has been shown to be associated with better cognitive development for children from vulnerable environments [29–31]. A novel aspect of this study was the examination of whether childcare attendance during the early childhood period moderated associations between MDS and children’s cognitive outcomes in kindergarten and grade 1. Contrary to previous research demonstrating that formal childcare attendance moderated associations between MDS and child internalizing behaviors [32–34], our results do not indicate that attending childcare is linked with better cognitive development in the context of MDS. This might be due to the fact that associations between MDS during the early childhood period and child behavior problems is larger than between MDS and children’s cognitive development (e.g., [16]). While attending childcare did not moderate associations between MDS and children’s cognitive development, attending formal and informal childcare was associated with higher scores on children’s cognitive development outcomes. However, these associations were no longer significant after adjusting for confounding variables.

We found that higher MDS was associated with lower scores on reading achievement in first grade for girls but not for boys. This contrasts with reports by Mensah and Kiernan [19] highlighting that boys tended to be more vulnerable to MDS for reading achievement. It is relevant to note that Mensah and Kiernan [19] evaluated the effect of concurrent maternal depression and did not study the contribution of MDS during the early childhood period. Furthermore, they used teacher-based assessments of academic achievement rather than objective tasks performed by the child. Teacher-based assessments might be negatively biased for boys of depressed mothers who could exhibit more disruptive behavior in school such as distractibility and inattention [78–80]. Our results however are consistent with a large-scale study that found that 16 years-old girls performed worse than boys at school in the context of MDS [23].

Although we found that MDS averaged over the early childhood years were negatively associated with children’s language development in kindergarten, and reading and mathematics achievement in first grade, effect sizes were small. Further, results failed to reach statistical significance once analyses were adjusted for confounding variables. Our finding of small effect sizes between MDS and children’s cognitive development is consistent with some previous studies [16, 17]. Maternal education as well as the child’s age in kindergarten and the number of months of schooling in first grade at the time of test administration were among those variables more strongly associated with child cognitive development. Attending school has previously been associated with gains in reading and mathematics performance and more specifically in the early years of primary school [62].

We found a small association between higher MDS and lower reading achievement scores in first grade for girls but no associations with academic readiness, language development and mathematic achievement for girls and boys. When evaluating the effect of MDS and economic deprivation on children’s academic readiness, Kiernan and Huerta [5] concluded that economic deprivation matters more for children’s academic readiness than MDS. This association was mainly mediated through cognitive stimulation. Furthermore, the association between economic deprivation and cognitive stimulation was much stronger than between MDS and cognitive stimulation. Consistent with these findings, Comaskey and colleagues [21] reported a much stronger association between family context and child academic readiness and achievement than between recurrent maternal depression and anxiety and such child outcomes. Our investigation is consistent with this work in suggesting that, within a community sample with elevated MDS (but not necessarily chronic or clinically significant symptoms), MDS does not appear to contribute significantly to child academic achievement and readiness over and above those risks factors associated with MDS such as lower maternal education and low income.

Recent studies have reported an association between MDS and lower academic achievement at 16 years old for boys and girls [81] with girls being even more vulnerable [23]. Pearson and colleagues [81] highlighted that attentional control measured at 8 years of age may mediate associations between MDS and academic achievement at 16 years old. This result indicates that different mechanisms could potentially explain associations between MDS and cognitive outcomes beyond parental engagement and parenting style. Furthermore, the relevance of such mechanisms or the effect sizes of associations between MDS and children’s cognitive development may depend on the child’s age and sex. Other mechanisms involved in the associations between MDS and children’s cognitive outcomes could include children’s working memory, peer social relationships, and externalizing behavior. Researchers have already highlighted associations between MDS and these potential mechanisms [12, 20, 82, 83] as well as between these mechanisms and academic achievement [84–86]. Further research is required to better understand the specific mechanisms, and the size and timing of their effects on associations between MDS and child academic achievement over the full academic journey.

Our study has the important advantage of being based on a large-scale longitudinal study covering the full early childhood period into school age with multiple independently assessed and well-validated measures of children’s cognitive development. Importantly, we considered various potential child, mother and family factors that might be associated with the context of MDS and children’s cognitive development. These risk factors could account for such associations and may also contribute a potential selection bias for the child to attend a specific childcare type. We also used weights to compensate for study attrition.

Despite the strengths of this large-scale study, we faced the following limitations. First, while we did not find that higher MDS averaged over the early childhood period were associated with lower scores on children’s cognitive outcomes in kindergarten and grade 1, our results may not generalize to families with chronically and clinically depressed mothers. Even if we identified a good proportion of mothers with elevated MDS, they would not necessarily meet criteria for clinical depression [72]. It is very possible that more pronounced effects would have emerged in a sample with a clinical diagnosis of depression. Second, although several variables were used to statistically adjust for a possible selection bias in childcare type, it is possible that some unidentified selection factors affected the results. Third, an emerging body of research links antenatal depression to children’s cognitive abilities [14, 87]. Since our measures of MDS were collected from the postnatal age of 5 months onwards, it was not possible to take this into consideration. Finally, we did not study mechanisms underlying associations between MDS and children’s cognitive development such as cognitive stimulation, the child’s motivation nor did we examine the role of the other parenting partner.

Overall, our findings indicate that factors such as maternal education, children’s age and the number of months of formal schooling at the time of outcome assessment, better explain children’s academic readiness and achievement at school entry than the average MDS score from 5 months to 5 years. Contrary to findings pertaining to emotional and behavioral outcomes, childcare did not appear to moderate associations between MDS and children’s cognitive development. Attending childcare might be more important for children from different at-risk backgrounds, such as those characterized by poverty or by more severe levels of depression.

## References

[1] Edwards CP, Susan M, Knoche L. Parent engagement and school readiness: Parent-child relationships in early learning. 2008.

[2] Hill NE. Parenting and academic socialization as they relate to school readiness: The roles of ethnicity and family income. Journal of Educational Psychology. 2001;93(4):686.

[3] Sohr-Preston SL, Scaramella LV. Implications of timing of maternal depressive symptoms for early cognitive and language development. Clinical Child and Family Psychology Review. 2006;9(1):65–83. 10.1007/s10567-006-0004-2 16817009

[4] Ahun MN, Côté SM. Maternal depressive symptoms and early childhood cognitive development: A review of putative environmental mediators. Archives of women's mental health. 2019;22(1):15–24. 10.1007/s00737-018-0870-x 29876681

[5] Kiernan KE, Huerta MC. Economic deprivation, maternal depression, parenting and children's cognitive and emotional development in early childhood 1. The British Journal of Sociology. 2008;59(4):783–806. 10.1111/j.1468-4446.2008.00219.x 19035922

[6] Stein A, Malmberg LE, Sylva K, Barnes J, Leach P, team F. The influence of maternal depression, caregiving, and socioeconomic status in the post‐natal year on children's language development. Child: care, health and development. 2008;34(5):603–12. 10.1111/j.1365-2214.2008.00837.x 18549438

[7] Campbell SB, Matestic P, von Stauffenberg C, Mohan R, Kirchner T. Trajectories of maternal depressive symptoms, maternal sensitivity, and children's functioning at school entry. Developmental psychology. 2007;43(5):1202 10.1037/0012-1649.43.5.1202 17723045

[8] Paulson JF, Dauber S, Leiferman JA. Individual and combined effects of postpartum depression in mothers and fathers on parenting behavior. Pediatrics. 2006;118(2):659–68. 10.1542/peds.2005-2948 16882821

[9] Cox A, Puckering C, Pound A, Mills M. The impact of maternal depression in young children. Journal of Child Psychology and Psychiatry. 1987;28(6):917–28. 10.1111/j.1469-7610.1987.tb00679.x 3436997

[10] Conners-Burrow NA, Bokony P, Whiteside-Mansell L, Jarrett D, Kraleti S, McKelvey L, et al Low-level depressive symptoms reduce maternal support for child cognitive development. Journal of Pediatric Health Care. 2014;28(5):404–12. 10.1016/j.pedhc.2013.12.005 24503001

[11] Lovejoy MC, Graczyk PA, O'Hare E, Neuman G. Maternal depression and parenting behavior: A meta-analytic review. Clinical Psychology Review. 2000;20(5):561–92. 10.1016/s0272-7358(98)00100-7 10860167

[12] Gueron-Sela N, Camerota M, Willoughby MT, Vernon-Feagans L, Cox MJ. Maternal depressive symptoms, mother-child interactions, and children’s executive function. Developmental psychology. 2018;54(1):71 10.1037/dev0000389 28933882 PMC5750080

[13] Field T. Postpartum depression effects on early interactions, parenting, and safety practices: a review. Infant Behavior and Development. 2010;33(1):1–6. 10.1016/j.infbeh.2009.10.005 19962196 PMC2819576

[14] Stein A, Pearson RM, Goodman SH, Rapa E, Rahman A, McCallum M, et al Effects of perinatal mental disorders on the fetus and child. The Lancet. 2014;384(9956):1800–19. 10.1016/S0140-6736(14)61277-0 25455250

[15] Liu Y, Kaaya S, Chai J, McCoy D, Surkan P, Black M, et al Maternal depressive symptoms and early childhood cognitive development: a meta-analysis. Psychological Medicine. 2017;47(4):680–9. 10.1017/S003329171600283X 27834159

[16] Brennan PA, Hammen C, Andersen MJ, Bor W, Najman JM, Williams GM. Chronicity, severity, and timing of maternal depressive symptoms: relationships with child outcomes at age 5. Developmental Psychology. 2000;36(6):759 10.1037//0012-1649.36.6.759 11081699

[17] Ahun MN, Geoffroy M-C, Herba CM, Brendgen M, Séguin JR, Sutter-Dallay L, et al Timing and chronicity of maternal depression symptoms and children's verbal abilities. The Journal of Pediatrics. 2017;190:251–7. 10.1016/j.jpeds.2017.07.007 28888562

[18] Letourneau NL, Tramonte L, Willms JD. Maternal depression, family functioning and children's longitudinal development. Journal of Pediatric Nursing. 2013;28(3):223–34. 10.1016/j.pedn.2012.07.014 22940454

[19] Mensah FK, Kiernan KE. Parents’ mental health and children’s cognitive and social development. Social Psychiatry and Psychiatric Epidemiology. 2010;45(11):1023–35. 10.1007/s00127-009-0137-y 19823757

[20] Kersten-Alvarez LE, Hosman CM, Riksen-Walraven JM, van Doesum KT, Smeekens S, Hoefnagels C. Early school outcomes for children of postpartum depressed mothers: comparison with a community sample. Child Psychiatry & Human Development. 2012;43(2):201–18. 22011810 10.1007/s10578-011-0257-y PMC3303034

[21] Comaskey B, Roos NP, Brownell M, Enns MW, Chateau D, Ruth CA, et al Maternal depression and anxiety disorders (MDAD) and child development: A Manitoba population-based study. PloS One. 2017;12(5):e0177065 10.1371/journal.pone.0177065 28542256 PMC5443487

[22] Murray L, Arteche A, Fearon P, Halligan S, Croudace T, Cooper P. The effects of maternal postnatal depression and child sex on academic performance at age 16 years: a developmental approach. Journal of Child Psychology and Psychiatry. 2010;51(10):1150–9. 10.1111/j.1469-7610.2010.02259.x 20840504

[23] Shen H, Magnusson C, Rai D, Lundberg M, Le-Scherban F, Dalman C, et al Associations of parental depression with child school performance at age 16 years in Sweden. JAMA Psychiatry. 2016;73(3):239–46. 10.1001/jamapsychiatry.2015.2917 26842307

[24] Grace SL, Evindar A, Stewart D. The effect of postpartum depression on child cognitive development and behavior: a review and critical analysis of the literature. Archives of Women’s Mental Health. 2003;6(4):263–74. 10.1007/s00737-003-0024-6 14628179

[25] Murray L, Hipwell A, Hooper R, Stein A, Cooper P. The cognitive development of 5‐year‐old children of postnatally depressed mothers. Journal of Child Psychology and Psychiatry. 1996;37(8):927–35. 10.1111/j.1469-7610.1996.tb01490.x 9119940

[26] Japel C, Tremblay RE, Côté S. Quality counts! Choices. 2005;11(5).

[27] Rigby E, Ryan RM, Brooks‐Gunn J. Child care quality in different state policy contexts. Journal of Policy Analysis and Management: The Journal of the Association for Public Policy Analysis and Management. 2007;26(4):887–908.

[28] Côté SM, Doyle O, Petitclerc A, Timmins L. Child care in infancy and cognitive performance until middle childhood in the millennium cohort study. Child Development. 2013;84(4):1191–208. 10.1111/cdev.12049 23331073

[29] Geoffroy MC, Côté SM, Giguère CÉ, Dionne G, Zelazo PD, Tremblay RE, et al Closing the gap in academic readiness and achievement: the role of early childcare. Journal of Child Psychology and Psychiatry. 2010;51(12):1359–67. 10.1111/j.1469-7610.2010.02316.x 20883519 PMC3283580

[30] Laurin JC, Geoffroy M-C, Boivin M, Japel C, Raynault M-F, Tremblay RE, et al Child care services, socioeconomic inequalities, and academic performance. Pediatrics. 2015;136(6):1112–24. 10.1542/peds.2015-0419 26598459

[31] Votruba-Drzal E, Coley RL, Koury AS, Miller P. Center-based child care and cognitive skills development: Importance of timing and household resources. Journal of Educational Psychology. 2013;105(3):821.

[32] Giles LC, Davies MJ, Whitrow MJ, Warin MJ, Moore V. Maternal depressive symptoms and child care during toddlerhood relate to child behavior at age 5 years. Pediatrics. 2011:peds. 2010–3119. 10.1542/peds.2010-3119 21669897

[33] Herba CM, Tremblay RE, Boivin M, Liu X, Mongeau C, Séguin JR, et al Maternal depressive symptoms and children’s emotional problems: Can early child care help children of depressed mothers? JAMA Psychiatry. 2013;70(8):830–8. 10.1001/jamapsychiatry.2013.1361 23784556

[34] Lee L-C, Halpern CT, Hertz-Picciotto I, Martin SL, Suchindran CM. Child care and social support modify the association between maternal depressive symptoms and early childhood behaviour problems: a US national study. Journal of Epidemiology & Community Health. 2006;60(4):305–10. 10.1136/jech.2005.040956 PMC2593413 16537346

[35] Radloff LS. Scale: A self-report depression scale for research in the general population. Journal of Clinical and Experimental Neuropsychology. 1997;19:340–56. 10.1080/01688639708403863 9268809

[36] Jenkins JM, Curwen T. Change in adolescents' internalizing symptomatology as a function of sex and the timing of maternal depressive symptomatology. Journal of the American Academy of Child & Adolescent Psychiatry. 2008;47(4):399–405. 10.1097/CHI.0b013e31816407db 18388768

[37] Poulin C, Hand D, Boudreau B. Validity of a 12-item version of the CES-D [Centre for Epidemiological Studies Depression scale] used in the National Longitudinal Study of Children and Youth. Chronic Diseases and Injuries in Canada. 2005;26(2–3):65. 16251012

[38] Vandell DL, Belsky J, Burchinal M, Steinberg L, Vandergrift N, NICHD Early Child Care Research Network. Do effects of early child care extend to age 15 years? Results from the NICHD study of early child care and youth development. Child development. 2010;81(3):737–56. 10.1111/j.1467-8624.2010.01431.x 20573102 PMC2938040

[39] National Institute of Child Health, Human Development Early Child Care Research Network. Child-care effect sizes for the NICHD Study of Early Child Care and Youth Development. American Psychologist. 2006;61(2):99–116. 10.1037/0003-066X.61.2.99 16478355

[40] Chew AL, Morris JD. Validation of the Lollipop Test: A diagnostic screening test of school readiness. Educational and Psychological Measurement. 1984;44(4):987–91.

[41] Venet M, Normandeau S, Letarte M-J, Bigras M. Mesure et évaluation: Les propriétés psychométriques du Lollipop. Revue de Psychoéducation. 2003.

[42] Dunn L, Dunn L. PPVT-R manual. Circle Pines, MN: American Guidance Service; 1981.

[43] Dunn LM, Theriault-Whalen C, Dunn L. Manual for échelle de vocabulaire en images Peabody. Toronto: Psycan 1993.

[44] Duncan GJ, Dowsett CJ, Claessens A, Magnuson K, Huston AC, Klebanov P, et al School Readiness and Later Achievement. Developmental psychology. 2007;43(6):1428 10.1037/0012-1649.43.6.1428 18020822

[45] Romano E, Babchishin L, Pagani LS, Kohen D. School readiness and later achievement: Replication and extension using a nationwide Canadian survey. Developmental Psychology. 2010;46(5):995 10.1037/a0018880 20822218

[46] Okamoto Y, Case R. II. Exploring the microstructure of children's central conceptual structures in the domain of number. Monographs of the Society for Research in Child Development. 1996;61(1‐2):27–58. 10.1111/j.1540-5834.1996.tb00536.x 8657168

[47] Gersten R, Jordan NC, Flojo JR. Early identification and interventions for students with mathematics difficulties. Journal of Learning Disabilities. 2005;38(4):293–304. 10.1177/00222194050380040301 16122059

[48] Kaufman AS, Kaufman NL. K-ABC—Kaufman assessment battery for children: Administration and scoring manual: American Guidance Service; 1983.

[49] Kaufman AS, Kaufman NL, editors. Batterie pour l'examen psychologique de l'enfant1993: ECPA.

[50] Duncan GJ, Gibson-Davis CM. Connecting child care quality to child outcomes: Drawing policy lessons from nonexperimental data. Evaluation Review. 2006;30(5):611–30. 10.1177/0193841X06291530 16966678

[51] Geoffroy M-C, Séguin JR, Lacourse É, Boivin M, Tremblay RE, Côté SM. Parental characteristics associated with childcare use during the first 4 years of life: Results from a representative cohort of Québec families. Canadian Journal of Public Health Revue Canadienne de Sante Publique. 2012;103(1):76 22338333 10.1007/BF03404073 PMC3283578

[52] Horta BL, Loret de Mola C, Victora CG. Breastfeeding and intelligence: a systematic review and meta‐analysis. Acta Paediatrica. 2015;104:14–9. 10.1111/apa.13139 26211556

[53] Antoniou E, Fowler T, Thiery E, Southwood T, Van Gestel S, Jacobs N, et al Intrauterine environment and cognitive development in young twins. Journal of Developmental Origins of Health and Disease. 2013;4(6):513–21. 10.1017/S2040174413000287 24924230

[54] Richards M, Hardy R, Kuh D, Wadsworth ME. Birth weight and cognitive function in the British 1946 birth cohort: longitudinal population based study. BMJ. 2001;322(7280):199–203. 10.1136/bmj.322.7280.199 11159613 PMC26584

[55] Bates JE, Freeland CAB, Lounsbury ML. Measurement of infant difficultness. Child Development. 1979:794–803. 498854

[56] Al-Hendawi M. Temperament, school adjustment, and academic achievement: existing research and future directions. Educational Review. 2013;65(2):177–205.

[57] Hotz VJ, Pantano J. Strategic parenting, birth order, and school performance. Journal of Population Economics. 2015;28(4):911–36. 10.1007/s00148-015-0542-3 26366045 PMC4565797

[58] Westerlund M, Lagerberg D. Expressive vocabulary in 18‐month‐old children in relation to demographic factors, mother and child characteristics, communication style and shared reading. Child: Care, Health and Development. 2008;34(2):257–66. 10.1111/j.1365-2214.2007.00801.x 18257795

[59] Bisanz J, Morrison FJ, Dunn M. Effects of age and schooling on the acquisition of elementary quantitative skills. Developmental Psychology. 1995;31(2):221.

[60] Cunningham A, Carroll J. Age and schooling effects on early literacy and phoneme awareness. Journal of Experimental Child Psychology. 2011;109(2):248–55. 10.1016/j.jecp.2010.12.005 21315371

[61] Cahan S, Davis D. A between-grade-levels approach to the investigation of the absolute effects of schooling on achievement. American Educational Research Journal. 1987;24(1):1–12.

[62] Luyten H, Merrell C, Tymms P. The contribution of schooling to learning gains of pupils in Years 1 to 6. School Effectiveness and School Improvement. 2017;28(3):374–405.

[63] Farrant BM, Zubrick SR. Parent-child book reading across early childhood and child vocabulary in the early school years: Findings from the Longitudinal Study of Australian Children. First Language. 2013;33(3):280–93.

[64] Dollaghan CA, Campbell TF, Paradise JL, Feldman HM, Janosky JE, Pitcairn DN, et al Maternal education and measures of early speech and language. Journal of Speech, Language, and Hearing Research. 1999;42(6):1432–43. 10.1044/jslhr.4206.1432 10599625

[65] Boivin M, Pérusse D, Dionne G, Saysset V, Zoccolillo M, Tarabulsy GM, et al The genetic‐environmental etiology of parents' perceptions and self‐assessed behaviours toward their 5‐month‐old infants in a large twin and singleton sample. Journal of Child Psychology and Psychiatry. 2005;46(6):612–30. 10.1111/j.1469-7610.2004.00375.x 15877767

[66] Canada S. Overview of survey instruments for 1994–95 data collection, cycle 1. Statistics Canada; 1995.

[67] Tabachnick BG, Fidell LS. Using multivariate statistics. Boston, MA, USA: Allyn & Bacon/Pearson Education; 2007.

[68] Byles J, Byrne C, Boyle MH, Offord DR. Ontario Child Health Study: reliability and validity of the general functioning subscale of the McMaster Family Assessment Device. Family process. 1988;27(1):97–104. 10.1111/j.1545-5300.1988.00097.x 3360100

[69] Desrosiers H, Boivin M, Des Groseilliers L. Concepts, Definitions and Operational Aspects,Part II–Data and Variables. Institut de la statistique du Québec, 2001 Contract No.: 12.

[70] Hayes AF, Montoya AK. A tutorial on testing, visualizing, and probing an interaction involving a multicategorical variable in linear regression analysis. Communication Methods and Measures. 2017;11(1):1–30.

[71] Seaman SR, White IR, Copas AJ, Li L. Combining multiple imputation and inverse‐probability weighting. Biometrics. 2012;68(1):129–37. 10.1111/j.1541-0420.2011.01666.x 22050039 PMC3412287

[72] Vilagut G, Forero CG, Barbaglia G, Alonso J. Screening for depression in the general population with the Center for Epidemiologic Studies Depression (CES-D): A systematic review with meta-analysis. PloS One. 2016;11(5):e0155431 10.1371/journal.pone.0155431 27182821 PMC4868329

[73] Naicker K, Wickham M, Colman I. Timing of first exposure to maternal depression and adolescent emotional disorder in a national Canadian cohort. PloS One. 2012;7(3):e33422 10.1371/journal.pone.0033422 22461893 PMC3312885

[74] Lanes A, Kuk JL, Tamim H. Prevalence and characteristics of postpartum depression symptomatology among Canadian women: a cross-sectional study. BMC Public Health. 2011;11(1):302. 21569372 10.1186/1471-2458-11-302 PMC3118237

[75] Warner RM. Applied statistics: From bivariate through multivariate techniques. Thousand Oaks, California, USA: Sage Publications, Inc.; 2008.

[76] Cohen J. Statistical power analysis for the behavioural sciences. Second ed New York: Hillsdale, NJ: erlbaum; 1988. 567 p.

[77] Berry D, Blair C, Willoughby M, Garrett-Peters P, Vernon-Feagans L, Mills-Koonce WR, et al Household chaos and children’s cognitive and socio-emotional development in early childhood: Does childcare play a buffering role? Early Childhood Research Quarterly. 2016;34:115–27. 10.1016/j.ecresq.2015.09.003 29720785 PMC5926246

[78] Beswick J, Willms JD, Sloat E. A comparative study of teacher ratings of emergent literacy skills and student performance on a standardized measure. Education. 2005;126(1):116.

[79] Heyder A, Kessels U. Do teachers equate male and masculine with lower academic engagement? How students’ gender enactment triggers gender stereotypes at school. Social Psychology of Education. 2015;18(3):467–85.

[80] Sinclair D, Murray L. Effects of postnatal depression on children's adjustment to school: Teacher's reports. The British Journal of Psychiatry. 1998;172(1):58–63. 9534834 10.1192/bjp.172.1.58

[81] Pearson RM, Bornstein MH, Cordero M, Scerif G, Mahedy L, Evans J, et al Maternal perinatal mental health and offspring academic achievement at age 16: the mediating role of childhood executive function. Journal of Child Psychology and Psychiatry. 2016;57(4):491–501. 10.1111/jcpp.12483 26616637 PMC4789117

[82] Hughes C, Roman G, Hart MJ, Ensor R. Does maternal depression predict young children’s executive function?–a 4‐year longitudinal study. Journal of Child Psychology and Psychiatry. 2013;54(2):169–77. 10.1111/jcpp.12014 23171379

[83] Goodman SH, Rouse MH, Connell AM, Broth MR, Hall CM, Heyward D. Maternal depression and child psychopathology: A meta-analytic review. Clinical Child and Family Psychology Review. 2011;14(1):1–27. 10.1007/s10567-010-0080-1 21052833

[84] Sabol TJ, Pianta RC. Patterns of school readiness forecast achievement and socioemotional development at the end of elementary school. Child Development. 2012;83(1):282–99. 10.1111/j.1467-8624.2011.01678.x 22103310

[85] Véronneau M-H, Vitaro F, Brendgen M, Dishion TJ, Tremblay RE. Transactional analysis of the reciprocal links between peer experiences and academic achievement from middle childhood to early adolescence. Developmental Psychology. 2010;46(4):773 10.1037/a0019816 20604601

[86] Van der Ende J, Verhulst FC, Tiemeier H. The bidirectional pathways between internalizing and externalizing problems and academic performance from 6 to 18 years. Development and Psychopathology. 2016;28(3):855–67. 10.1017/S0954579416000353 27427810

[87] Milgrom J, Holt C, Bleker L, Holt C, Ross J, Ericksen J, et al Maternal antenatal mood and child development: an exploratory study of treatment effects on child outcomes up to 5 years. Journal of Developmental Origins of Health and Disease. 2018:1–11. 10.1017/S2040174418000739 30303063

